# GNSS visibility and performance implications for the GENESIS mission

**DOI:** 10.1007/s00190-023-01784-4

**Published:** 2023-10-31

**Authors:** Oliver Montenbruck, Peter Steigenberger, Steffen Thoelert, Daniel Arnold, Grzegorz Bury

**Affiliations:** 1 German Space Operations Center, Deutsches Zentrum für Luft- und Raumfahrt, Münchener Str. 20, 82234 Weßling, Germany; 2 Institute of Communications and Navigation, Deutsches Zentrum für Luft- und Raumfahrt, Münchener Str. 20, 82234 Weßling, Germany; 3 Astronomical Institute, University of Bern, Sidlerstrasse 5, 3012 Bern, Switzerland; 4 Institute of Geodesy and Geoinformatics, Wroclaw University of Environmental and Life Sciences, Grunwaldzka 53, 50-357 Wroclaw, Poland

**Keywords:** GENESIS, Gain pattern, Transmit power, GPS, Galileo, Antenna

## Abstract

The GENESIS mission prepared for launch in 2027 integrates the four space-geodetic techniques on a single spaceborne platform in medium Earth orbit. With its unique observations and alternative tie concepts, the mission aims to contribute to an improved accuracy and homogeneity of future terrestrial reference system realizations. To assess the expected contribution of Global Navigation Satellite System (GNSS) tracking, a comprehensive GNSS coverage analysis is performed based on detailed link-budget simulations, taking into account the best available gain patterns and signal-specific transmit power estimates derived for this work from measurements of a high-gain dish antenna. The benefit of different receiver antenna concepts for the GENESIS spacecraft is assessed, and it is demonstrated that a single-antenna system with either a nadir-looking or side-looking boresight is a viable alternative to the dual-antenna configuration considered in initial mission studies. Compared to terrestrial users and missions in low Earth orbit, GENESIS will collect GNSS signals transmitted at up to two times larger off-boresight angles. Only limited information on the actual transmit antenna phase patterns is presently available in this region, which hampers a quantitative assessment of the expected measurement and orbit determination accuracy. As such, a comprehensive release of manufacturer calibrations is encouraged for all blocks of GPS and Galileo satellites. In parallel, a need for in-flight characterization and calibration of the GNSS transmit antennas for off-boresight angles of up to $30^\circ$ using observations of the GENESIS mission itself is expected. The impact of such calibrations on the overall quality of terrestrial reference frame parameters will need to be assessed in comprehensive simulations of global GNSS network solutions with joint processing of terrestrial and GENESIS GNSS observations.

## Introduction

GENESIS (Delva et al. 2023; Ventura-Traveset 2022) is the initial mission of a cross-disciplinary GNSS/NAV Science Programme proposed by the European Space Agency (ESA). It is currently planned for launch in 2027 and will offer the first-ever co-location of the four space-geodetic techniques in space. GENESIS builds on earlier mission concepts such as the Geodetic Reference Antenna in Space mission (GRASP; Bar-Sever et al. 2009) and the E-GRASP/Eratosthenes mission proposed for ESA’s Earth Explorer Mission EE-9 (Biancale et al. 2017; Pollet et al. 2023). Like these, GENESIS aims to improve the accuracy and stability of the terrestrial reference frame (TRF) at a targeted performance of 1 mm and 0.1 mm/y, respectively (Gross et al. 2009). To achieve these goals, the GENESIS spacecraft will host a Global Navigation Satellite System (GNSS; Betz 2015) receiver, a Doppler Orbitography and Radiopositioning Integrated by Satellite (DORIS; Auriol and Tourain 2010) receiver, a laser retro-reflector array (LRA) for Satellite Laser Ranging (SLR; Combrinck 2010), and, finally, a beacon for Very Long Baseline Interferometry (VLBI; Schuh and Böhm 2013). Compared to other space missions, such as Sentinel-3A/B (Fletcher 2012) and Sentinel-6A (Donlon et al. 2021), which already offer a GNSS, DORIS, and SLR co-location, the addition of VLBI offers a unique opportunity to improve the link between the terrestrial and the celestial reference frame.

While the ground visibility of Earth orbiting satellites, and thus, the expected SLR, VLBI, and DORIS coverage, increases with altitude, the opposite is generally true for GNSS tracking with a spaceborne receiver (Stanton et al. 2006). Based on early mission studies, an altitude of $h={6000}~\textrm{km}$ has been proposed for GENESIS as a compromise between these conflicting requirements (Delva et al. 2023; Ventura-Traveset 2022). From a GNSS perspective, this clearly leaves the so-called Terrestrial Service Volume, which provides established performance and availability commitments for land, air, and space users up to a peak altitude of 3000 km (DOD 2020). Users in the Space Service Volume (SSV; UNOOSA 2021), in contrast, experience notably different visibility conditions and are not yet protected by comprehensive service performance commitments of the GNSS providers. To cope with these limitations, use of a dual-antenna system offering joint tracking with a zenith-looking and a nadir-looking antenna is considered in the current baseline of the GENESIS design.

This study investigates the conditions and expected measurement performance of GPS and Galileo tracking from the GENESIS satellite. In a first step, the geometric visibility for satellites with the zenith and nadir antennas is discussed in Sect. 2. As an input for the detailed link-budget analysis, transmit antenna gain patterns for the various blocks of GPS and Galileo satellites are compiled in Sect. 3 and complemented with signal-specific transmit power values derived from the analysis of high-gain antenna measurements. Based on these, the expected availability of GNSS tracking in the GENESIS orbit is assessed in Sect. 4 for the individual constellations, signals and antennas, while Sect. 5 characterizes the expected positioning performance through a dilution-of-precision analysis and a discussion of expected contributions to the range error budget. A summary of key findings and relevant conclusions are presented in Sect. 6.

**Fig. 1:**
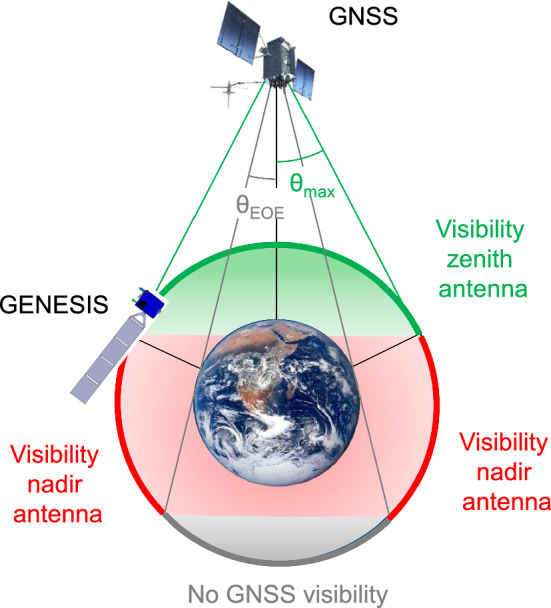
Schematic view of GNSS visibility from a satellite at 6000 km altitude

## Geometric visibility conditions

Based on the early mission design described in Ventura-Traveset (2022), GENESIS will fly in a polar orbit with a height of $h={6000}~\textrm{km}$ and an orbital inclination of 95.5$^\circ$. The purely geometric conditions of GNSS visibility at the GENESIS altitude are illustrated in Fig. 1. For a GNSS satellite with orbital radius $r_\text {GNSS}$, the edge-of-Earth (EOE) half-beamwidth amounts to

1 $$\begin{aligned} \theta _\text {EOE}=\arcsin (R_\oplus /r_\text {GNSS}) \end{aligned}$$

with $R_\oplus$ denoting the Earth radius, while the GENESIS orbit extends to peak off-boresight angles of

2 $$\begin{aligned} \theta _\text {max}=\arcsin ((R_\oplus +h)/r_\text {GNSS}). \end{aligned}$$

These amount to roughly $28^\circ$ for GPS and $25^\circ$ for Galileo.

With a zenith antenna alone, a GNSS satellite can be tracked, if the orbital position of GENESIS is located in the green region characterized by $\theta <\theta _\text {max}$ and GNSS-to-GENESIS distances of less than $\sin (\theta _\text {max})\!\cdot \!r_\text {GNSS}$. Nadir antenna tracking is available in the red region ($\theta _\text {EOE}<\theta <\theta _\text {max}$), while Earth blockage inhibits tracking in the gray region on the far side of the Earth as seen from the GNSS satellite. For small angles of the Earth-GNSS vector relative to the orbit plane, alternating phases of zenith-antenna tracking, nadir-antenna tracking and Earth blockage arise throughout the orbit, while higher angles result in continuous nadir-antenna visibility.

It may be noted that tracking with the nadir antenna implies larger distances and thus, reduced received power levels compared to the zenith antenna. Also, the comparatively good geometric coverage offered by a dual-antenna system should not obscure the fact that most of the tracking takes place outside the Earth coverage region of the GNSS main lobe for which the respective transmit antenna characteristics have been optimized. In particular, antenna gains at off-boresight angles $\theta >\theta _\text {EOE}$ experience a rapid and notable decrease, which poses obvious constraints to the feasibility and quality of the GNSS tracking in the GENESIS orbit. Likewise, only limited public information is available concerning phase and group delay variations outside the Earth coverage zone.

**Fig. 2:**
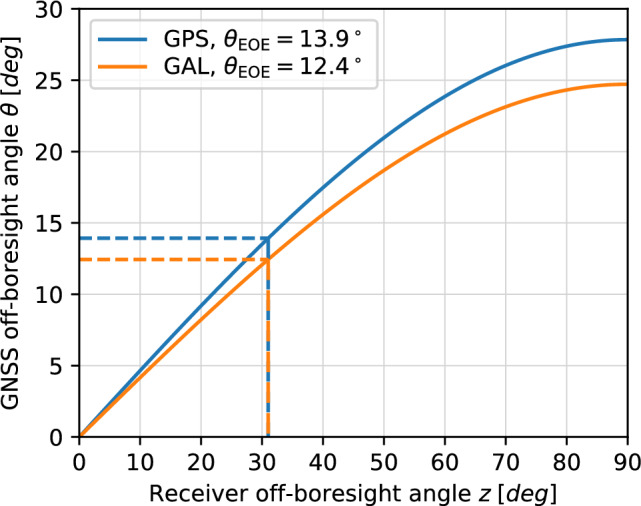
GNSS visibility for the GENESIS mission (solid lines). Dashed lines mark the edge-of-Earth coverage zone of the GNSS transmit antenna, and, equivalently, the Earth blockage region for the nadir-oriented receiver antenna

The relation between the off-boresight angles of the transmit and receive antennas is illustrated in Fig. 2 for the two constellations. In view of the higher altitude, a slightly smaller Earth coverage beamwidth applies for Galileo than for GPS. The relation between off-boresight angles of the transmit and receiver antenna is equally valid for both the zenith-looking antenna and the nadir-looking receive antenna. For GNSS tracking with the zenith-antenna, off-boresight angles *z* of less than about $30^\circ$ correspond to the EOE transmit antenna cone, which is covered and well-characterized by terrestrial GNSS observations. Observations at larger zenith angles, in contrast, would still require proper characterization of the transmit antennas by the GENESIS mission itself, unless fully representative factory calibrations can be made available by all relevant GNSS providers.

At the same time, observations in this region will be affected by a notable reduction in the received signal power that is further discussed in Sect. 3. For the nadir looking antenna, substantially different visibility conditions apply. Here, GNSS signals are blocked for small off-nadir angles up to the EOE limit, allowing only observations in a region of $z>31^\circ$ or, equivalently, transmit off-boresight angles above $\theta =13.9^\circ$ (GPS) or $\theta =12.4^\circ$ (Galileo). From a practical point of view, a slightly larger Earth obscuration angle of about $31.2^\circ$ will be assumed in the GENESIS data processing to exclude Earth-grazing observations that are affected by path delays, bending, and attenuation in the neutral atmosphere. Aside from the aspects already discussed above, nadir antenna observations in this off-boresight angle range are also affected by an increased receiver-transmitter distance as well as possible interference or noise from ground-based radio frequency transmissions.

**Table 1:** Overview of GNSS transmit antenna gain patterns for different types of GPS and Galileo satellites

Block	Source and description	Grid
GPS Block IIR-A, GPS Block IIR-B, GPS Block IIR-M	Block-mean values of manufacturer-calibrated L1 and L2 directivity patterns and gain corrections from Marquis (2014, 2015); Marquis and Reigh (2015); Marquis (2016)	$2^\circ \times 10^\circ$, $\theta \le 90^\circ$
GPS Block IIF	Block-mean inner pattern ($\theta \le 23^\circ$) for L1, L2, L5 from conical cut data of Igwe (2023), outer L1 pattern (block-mean) from Donaldson et al. (2020); outer L2 and L5 patterns (block- and azimuth-mean) from great-circle cuts of Igwe (2023)	$2^\circ \times 10^\circ$, $\theta \le 90^\circ$
GPS Block III	Block-mean values of manufacturer-calibrated L1, L2, and L5 directivity patterns from Fisher (2022a, 2022b); approximate gain correction factor ${-1}~\textrm{dB}$ from IIR-B/IIR-M satellites	$2^\circ \times 10^\circ$, $\theta \le 90^\circ$
Galileo IOV	Digitized high-band (E1) and low-band off-boresight-angle-dependent patterns of enhanced NAVANT from Monjas et al. (2010); low-band pattern scaled from 1237 MHz to E5	$1^\circ$, $\theta \le 30^\circ$
Galileo FOC	Digitized off-boresight-angle-dependent high-band (E1) and low-band patterns from Valle et al. (2006); low-band pattern scaled from 1279 MHz to E5	$1^\circ$, $\theta \le 30^\circ$

The specified resolution $\Delta \theta$ for off-boresight-angle-dependent, azimuth-averaged patterns or $\Delta \theta \times \Delta A$ for off-boresight-angle- and azimuth-dependent patterns refers to the resolution employed for the current study and involves a down-sampling for some of the original data sets

## Signal modeling

The modeling of GENESIS GNSS tracking and visibility conditions makes use of established link-budget models along with antenna patterns and transmit power values that are discussed in the present section.

### Link budget

Following Misra and Enge (2011), the effective signal power $P_\text {R}$ experienced by a receiver at a distance *r* from a GNSS satellite is described by the relation

3 $$\begin{aligned} P_\text {R} = \frac{P_\text {T}G_\text {T}G_\text {R}}{L_\text {R}}\left( \frac{\lambda }{4\pi r}\right) ^2 , \end{aligned}$$

when including the receiver antenna gain ($G_\text {R}$) and receiver losses ($L_\text {R}$). Here, $P_\text {T}$ and $G_\text {T}$ denote the satellite transmit power and antenna gain. The term in brackets describes the contribution of the free-space loss, which depends on the ratio of the distance and the wavelength $\lambda$ of the respective signal. The tracking conditions and measurement noise in the receiver are characterized by the ratio $C/N_0$ of the signal power and the noise power $N_0$ in a 1 Hz bandwidth, which is commonly expressed as the product $N_0=kT_\text {eq}$ of the Boltzmann constant ${1.38 \times 10^{-23}}{J/K}$ and the equivalent noise temperature $T_\text {eq}$. In a logarithmic form, the resulting carrier-to-noise-power density ratio can thus be modeled as

4 $$\begin{aligned} (C/N_0)_\text {[dBHz]}= & {} P_\text {T,[dBW]} + G_\text {T,[dB]} + G_\text {R,[dB]}\nonumber \\{} & {} - 20\log _{10}(4\pi r/\lambda ) \nonumber \\{} & {} + 228.6 - T_\text {eq,[dBK]} - L_\text {R,[dB]}. \end{aligned}$$

The various link-budget contributions and their respective values are further discussed in the following subsections.

Equation (4) is applicable for all open GNSS signals that enable tracking with a known ranging code including the GPS L1 C/A, L2C, and L5 signals as well as the Galileo E1 Open Service and E5a, E5b, and E5ab signals. It would likewise apply for dedicated military GPS receivers enabling direct tracking of the GPS P-code after decryption of the transmitted Y-code. Civil tracking of the military P(Y)-code, on the other hand, requires the use of semi-codeless tracking schemes that suffer from signal-strength-dependent squaring losses (Woo 2000; Montenbruck et al. 2006). Considering the Z-tracking variant of Silvestrin and Cooper (2000) that is most widely used in European GNSS receivers for space applications, the carrier-to-noise density ratio for semi-codeless tracking on both L1 and L2 can approximately be described as a function of the $C/N_0$ values for unencrypted P-code tracking on the two frequencies by the relation

5 $$\begin{aligned}{} & {} (C/N_0)_\text {P(Y),L1/L2,[dBHz]}\nonumber \\{} & {} \quad \approx (C/N_0)_\text {P,L1,[dBHz]} + (C/N_0)_\text {P,L2,[dBHz]} - 55.6 \end{aligned}$$

for representative signal strengths of $(C/N_0)_\text {P,Li} \lesssim {50}~\textrm{dBHz}$ (J. Christensen, RUAG, priv. comm.). Similar relations with slightly different losses apply for other semi-codeless schemes as discussed in Woo (2000).

**Fig. 3:**
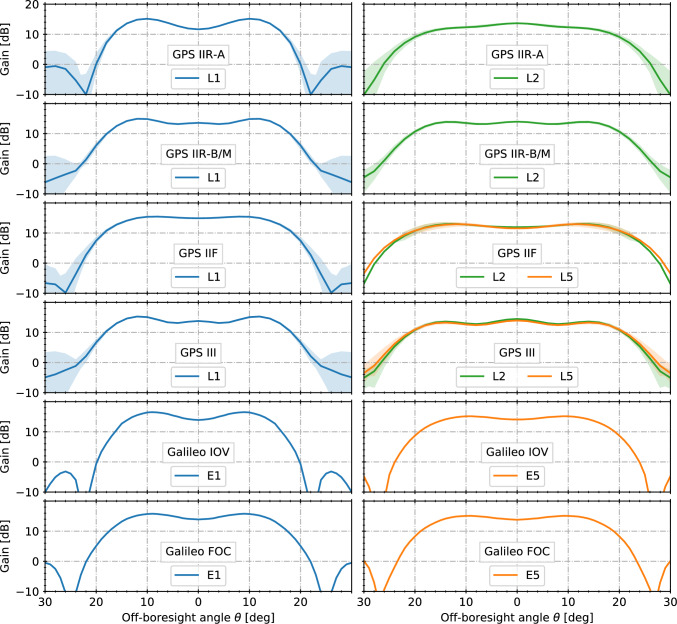
Azimuth-averaged gain patterns of different types of GPS and Galileo satellites in the lower L-band (L1/E1; left) and upper L-band (L2, L5, E5; right) as a function of the off-boresight angles. Where available, shaded areas describe the gain pattern variation across different azimuth angles

### Transmit antenna gain

Consistent with the dominating use of GNSSs for terrestrial and near-Earth navigation, GNSS transmit antennas exhibit a high directivity that focuses the transmitted energy on the Earth’s surface. At representative half-beamwidths of $20^\circ$, the peak antenna gains amount to roughly 15 dB and allow to ensure the required minimum receiver power levels with transmit powers in the range of 10–100 W (Spilker Jr. 1996). To partly compensate the varying distance across the surface of the Earth and the associated variations of free-space losses, it is furthermore common practice to employ an M-shaped gain pattern (Brumbaugh et al. 1976) with a slightly lower gain in boresight-direction than toward the EOE for all GNSS transmit antennas. This particular shape is achieved by combining an inner (wide-beam) and an outer (narrow-beam) ring of antenna elements with a $180^\circ$ phase shift. While GPS uses identical sets of antenna elements for all frequencies, causing a wider pattern for L2 and L5 as compared to L1, more balanced beamwidths are achieved in the case of Galileo by a larger number and band-specific allocation of individual transmit antenna elements.

A summary of gain pattern data used for the present study is given in Table 1. Comprehensive gain pattern information is presently available for the GPS Block IIR satellites with legacy (designated as IIR-A) and modernized antenna panel (IIR-B), as well as Block IIR-M and GPS III satellites, for which ground-based calibrations have been released through the satellite manufacturer (Lockheed Martin) and the GPS program. The satellite-specific calibrations cover both the main lobe and sidelobes at the individual frequencies for off-boresight angles up to $90^\circ$. In the case of GPS III, published data are limited to the directivity patterns, whereas gain corrections factors are presently lacking. Given the similarity of the respective antenna panels, an approximate gain correction factor of −1 dB has been applied for L1, L2, and L5 patterns of GPS III in analogy with the Block IIR-B/IIR-M values of Marquis and Reigh (2015).

For the GPS IIF satellite antennas, manufacturer calibrations have only been released in May 2023 by Boeing and the GPS authorities (Igwe 2023). Most notably, these calibrations include gain patterns for the L1, L2, and L5 frequency based on a series of conic scans for off-boresight angles of up to $23^\circ$. In addition, a sparse set of great circle scans up to $\theta =90^\circ$ is provided for selected IIF satellites. The latter data do not enable a realistic description of individual sidelobes but could at least be used to establish block- and azimuth-averaged L2 and L5 patterns for use beyond $\theta >23^\circ$ in the present GNSS visibility study. In the case of the L1 frequency, azimuth- and off-boresight-angle-dependent gain patterns outside the Earth coverage zone have been measured in the frame of the GPS Antenna Characterization Experiment (ACE) onboard a geostationary satellite (Donaldson et al. 2020). These provide a more realistic description of sidelobe gain variations than the Boeing data and have therefore been used to complement the manufacturer calibrations of the antenna mainlobe beyond the $23^\circ$ limit.

The current Galileo constellation is mainly composed of Full Operational Capability (FOC) satellites, but also contains a small number of In-Orbit Validation (IOV) satellites that have been built by a different manufacturer and make use of a different transmit antenna design. So far, no gain pattern calibrations have been released for either IOV or FOC satellites by the Galileo project. In view of this limitation, we refer to partial information made available by the antenna manufacturers in the early development stage. While this information might not be fully representative of the final antenna design, it represents the best available public information and is expected to provide a suitable basis for the purpose of Galileo link-budget computations in GNSS visibility studies. Specifically, we made use of gain patterns for the lower and upper L-band presented in Monjas et al. (2010) and Valle et al. (2006) for IOV and FOC antennas, respectively. In the latter case, we assume that the “Enhanced NAVANT”, which describes a follow-on design of the original GIOVE-A antenna and shares the same layout as the FOC antenna, is representative of the final FOC antenna. Gain patterns for $\theta \le 30^\circ$ were digitized from illustrations in the respective publications considering only the average nadir-angle variation but ignoring azimuth dependencies. The latter are quite notable outside the main lobe region, but cannot be extracted from the graphical representation with adequate confidence. In the case of the lower L-band data, which refer either to E6 (Valle et al. 2006) or an intermediate frequency between E5 and E6 (Monjas et al. 2010), a scaling of $\sin (\theta )$ by the corresponding wavelength ratio was used to obtain the desired gain patterns at the E5 frequency.

The block-specific patterns for the upper and lower L-band are illustrated in Fig. 3. In the L1 or E1 band, all antennas show a rapid gain role-off outside the EOE region with deep minima at off-boresight angles of about 20$^\circ$–25$^\circ$, while the L2 and L5/E5 patterns are generally wider. In general, the main-lobe beamwidth in the upper L-band is smaller than the geometric visibility region for GENESIS, and the availability of dual-frequency tracking is therefore expected to be driven by the L1 and E1 signals.

### Transmit power

Next to the gain patterns of the GNSS satellites, the modeling of received signal powers depends on knowledge of the corresponding transmit powers. In principle, transmit power values for individual signals can be inferred from specifications of the minimum received signal power made by the GNSS providers as part of their performance commitments. However, it is difficult to use this information in practice to derive the actual transmit powers in view of conservative link budgets and notable design margins of the manufacturers (Betz 2010; Marquis 2016). Satellite-specific L1 and L2 transmit power values for individual satellites are provided in Wu (2002) but limited to Block IIR-A satellites that cover only a small subset of today’s GPS constellation. A wider set of satellite types is covered by ground received power measurements of Wang et al. (2018), but the respective data apply only for the GPS L1 band.

We therefore made use of block-average transmit power measurements obtained for representative satellites of the current GPS and Galileo constellation with the 30 m high-gain dish antenna of DLR’s signal-monitoring facility in Weilheim, Germany (Thoelert et al. 2009). Following the approach of Steigenberger et al. (2018), band-specific transmit powers for the L1/L2/L5 and E1/E5a frequencies were obtained that comprise the total power of all signals modulated on the respective carriers. These were further split into the contribution of individual signals making use of known or measured power sharing for the individual signal components and, where applicable, inter-modulation products (Betz 2015; Partridge and Dafesh 2001). Where required, actual power sharing ratios for specific satellite types and signal modulations were obtained from in-phase-quadrature signal samples or signal spectra collected with the high-gain antenna (Thoelert et al. 2018). In the case of GPS III, it may be noted that the military M-code contributes to the received L1 and L2 signal powers but is transmitted from a separate antenna (Thoelert et al. 2019). For these satellites, the M-code contribution to the total power was separated using a modeled gain pattern of the M-band antenna and a least-squares fit of the modeled and observed effective isotropic radiation power (EIRP) variation over off-boresight angle.

For validation and further refinement of the resulting signal- and block-specific transmit power values, the expected $C/N_0$ values were modeled for the PODRIX GNSS receiver onboard the Sentinel-6A satellite. The multi-frequency, dual-constellation receiver offers tracking of the L1 C/A, L1 P(Y), L2-CL, L2 P(Y), and L5-Q for GPS, as well as E1-C and E5a-Q of Galileo (Montenbruck et al. 2021). The receiver uses a patch-excited cup (PEC; Öhgren et al. 2011) antenna and is considered here as a reference for the future GENESIS GNSS receiver. Band-specific gain patterns of the PEC antenna have been made available as part of the Sentinel-6A instrument calibration data base. As a complement, and to cope with a receiver-specific bias in the $C/N_0$ for the Open Service E1 signal of Galileo, measurements from a PolaRx5 receiver at the MAO0 station of the International GNSS Service (IGS; Johnston et al. 2017) were used along with the in-house-calibrated gain patterns of the employed Leica AR25 antenna.

**Table 2:** Block- and signal-specific transmit power values adopted for the GENESIS link-budget analysis

GPS	L1 C/A	L1 P(Y)	L2-CL	L2 P(Y)	L5-Q
IIR-A	14.5	12.0		10.0	
IIR-B	14.5	12.5		10.0	
IIR-M	14.5	12.0	12.0	12.0	
IIF	14.0	11.5	13.5	12.0	16.0
III	13.5	11.0	13.5	11.0	16.0
Galileo	E1-C	E5a-Q			
IOV-1/2	10.5	9.0			
IOV-3	9.0	8.0			
FOC	14.5	12.5			

All values in dBW

The resulting transmit power values for individual satellite types and the signals of interest are summarized in Table 2. Given prevailing uncertainties or inconsistencies in the employed gain patterns and received power measurements, the individual values are believed to exhibit a representative accuracy of about 1 dB or 25%, which appears adequate for the study purpose. In the case of Galileo, different power levels are given for the three IOV satellites that reflect the individual power level reductions after a failure of the IOV-4 satellite. For GPS P(Y) code, the power levels refer to normal transmit power and ignore the power increase in up to +6 dB observed in recent years for certain periods or parts of their orbit under flex power operations (Esenbuğa et al. 2023). The adoption of nominal P(Y) power levels for the present simulations is motivated by the fact that flex power operations are likely to become obsolete before the launch of GENESIS due to the improving availability of M-code for use by US military forces.

### Receiver antenna gain

**Fig. 4:**
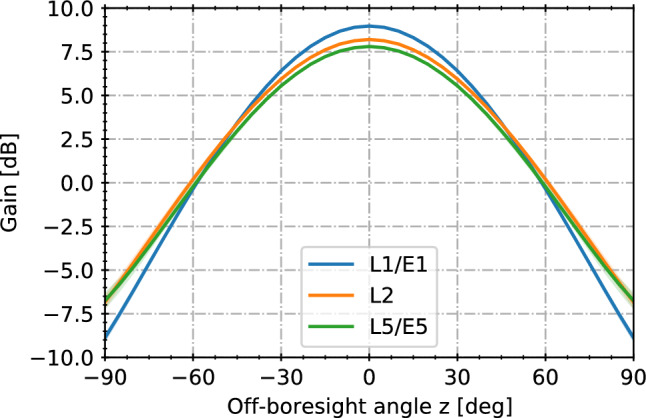
Multi-frequency antenna gain pattern adopted for the GENESIS link-budget simulations

Similar to other space missions requiring high-precision orbit determination for remote sensing and geodesy, we assume use of a chokering antenna with a moderately focused antenna pattern. The specific gain pattern adopted for this study is shown in Fig. 4. It represents the GNSS antenna design used on the Swarm and Sentinel spacecraft (Öhgren et al. 2011) and exhibits peak gains of about 8–9 dB along the boresight direction and a gain roll-off of 15–18 dB toward the antenna horizon. Slightly lower peak gains and a moderately wider beamwidths apply for lower L-band frequencies as opposed to L1/E1.

It may be noted that the specific antenna type adopted here offers low phase variations, a high level of multipath suppression, and good sensitivity for tracking of GNSS main-lobe signals with the GENESIS zenith antenna, but is not necessarily optimized for tracking weak signals at mid- and low elevations. Possible alternatives may include helix antennas (Dilssner et al. 2006) with a less focused gain pattern or the use of three-dimensional choke ring designs (Kunysz 2003) as employed in selected terrestrial antennas. However, analyses and trade-off of alternative antenna designs with optimized gain and multipath characteristics for the GENESIS mission are beyond the scope of the current study and left to the on-going mission design phase.

### Receiver system noise and implementation losses

As described by (4), the achieved $C/N_0$ for a given received signal power is determined by the equivalent system noise temperature $T_\text {eq}$ and additional implementation losses $L_\text {R}$ in the receiver. For a cascaded radio frequency system composed of different passive elements (cables, connectors, filters, splitters, etc.) and active elements (amplifiers), the equivalent noise temperature can be described by Friis’s equation (Misra and Enge 2011; van Diggelen 2009), provided that the individual losses, gains, and noise figures are known.

The antenna constitutes the first noise source in the receiver system. The associated noise temperature $T_\text {A}$ results from contributions of the observed sky background (4 K) and possible Earth radiation in the field of view (290 K) as well as radiation from the host platform received through the antenna backplane. For terrestrial receivers, a representative value of ${130}~\textrm{K}$ is given by Spilker Jr. (1996), while a notably smaller value $T_\text {A}={34}~\textrm{K}$ has been adopted by Winternitz et al. (2019) for high-altitude spacecraft based on flight data of the Magnetospheric Multiscale (MMS) mission.

Subsequent contributions are dominated by cable and connector losses prior to the low noise amplifier (LNA) and the LNA noise figure. Assuming values of 1 dB and 1.5 dB, respectively, an equivalent system temperatures at the level of $T_\text {eq}={300}~\textrm{K}$ or ${24.7}~\textrm{dBK}$ are attained (van Diggelen 2009), which closely, but coincidentally, agrees with representative values of the ambient receiver temperature. Taking into account the additional implementation losses with a representative size of 2 dB (van Diggelen 2009), the resulting carrier-to-noise density ratio is reduced by 26.7 dB compared to a loss-less system at 0 K noise temperature.

The above values outline the expected range of noise values in general GNSS receiver systems but can only provide a coarse reference for the specific case of GENESIS. Given the restricted availability of public information on the noise characteristics of actual space receiver systems, we therefore performed an empirical calibration of the combined noise temperature and losses for the Sentinel-6A GNSS subsystem as a basis for the present study. Based on the comparison of observed $C/N_0$ values for the various GPS and Galileo signals with simulated values for the previously described gain patterns and transmit power levels, an aggregate “noise factor”

6 $$\begin{aligned} T_\text {eq,[dBK]} + L_\text {R,[dB]} = 28.5 \end{aligned}$$

with an expected uncertainty of ${\pm 1\,\, \hbox {dB}}$ has been obtained in this way.

While the individual contributions are not known with certainty, this noise factor can be understood by an equivalent system temperature of roughly $T_\text {eq}={450}~\textrm{K}$ and additional implementation losses of $L_\text {R}={2}~\textrm{dB}$. Compared to the sample noise budget discussed above, a slightly higher system noise level is obtained, which reflects the actual conditions of an established multi-frequency GNSS system for space applications based on a passive antenna, detached low-noise amplifier, and additional harness losses. The noise factor (6) is therefore considered as representative for the envisaged use case and adopted as a reference value for the subsequent simulations.

Even though all effort has been made to come up with realistic assumptions for the GENESIS link-budget computations, it is obvious that actual receiver and antenna characteristics are unknown in the present project stage. Also, conservative pre-mission performance assumptions were exceeded in various high-altitude missions such as MMS (Lulich et al. 2012) and the geostationary LUCAS relay satellite (Matsumoto et al. 2022), which showed a notably better than expected GNSS tracking coverage. To cope with these uncertainties, selected tests are also conducted with $\pm {3}~\textrm{dB}$ variations of the reference noise factor (6).

**Fig. 5:**
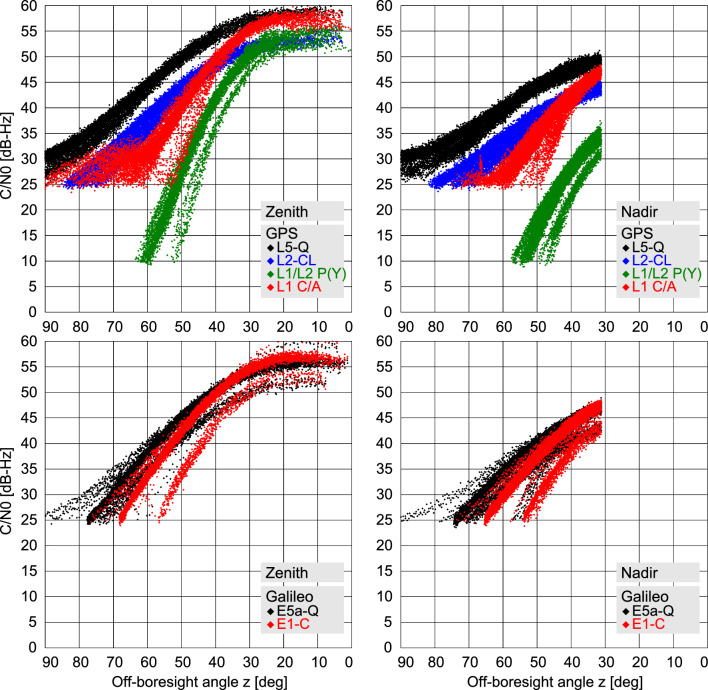
$C/N_0$ values of GPS (*top*) and Galileo (*bottom*) satellites tracked with the zenith (*left*) and nadir (*right*) antenna based on link-budget simulations for the GENESIS mission

## Tracking and visibility simulation

Based on the gain, power, and noise models described in the previous section, tracking and visibility conditions were simulated considering both a zenith pointing and a nadir pointing antenna. A 24-h data arc on 1 January 2023 was considered, making use of the GPS and Galileo constellation available on this date. This comprised various unhealthy satellites transmitting valid signals and considered in precise orbit and clock products of the IGS. Overall, 31 GPS and 26 Galileo including the two Galileo satellites E201 and E202 were included in the simulation. For GENESIS, a polar orbit with a $95.5^\circ$ inclination as discussed in Ventura-Traveset (2022) was assumed. While no decision on the actual orbit has been taken, yet, a highly inclined orbit will provide optimum Earth coverage and is compatible with launch opportunities for common remote sensing satellites.

Since Sun-synchronous circular orbits cannot be realized at the 6000 km altitude selected for the GENESIS mission, the local time of the ascending node (LTAN) will inevitably vary throughout the mission (Vallado 2001). Without loss of generality, an orbit with a 14-h LTAN has been adopted for our simulation, implying a Sun elevation close to 30$^\circ$ above the orbital plane. Along with this, a yaw steering similar to that of common GNSS satellites is assumed for GENESIS, which maintains a nadir-pointing $+z$-axis, while orienting the solar panel rotation axis ($\pm y$) perpendicular to the Sun (Hugentobler and Montenbruck 2017) to maximize the available energy. The specific choice of this attitude model is of no relevance for modeling the GNSS visibility of the zenith and nadir antennas, but affects the field of a side-looking antenna that will later be considered as an alternative to the currently considered dual-antenna configuration. Furthermore, knowledge of the spacecraft body and solar panel orientation will be important for a realistic modeling of non-gravitational forces in future orbit determination studies. For the LTAN adopted here and the associated $\beta$-angle, the spacecraft $+x$-axis is rotated by yaw angles of $\Psi =-30^\circ$ to $\Psi =-150^\circ$ relative to the along-track direction.

**Table 3:** Percentage of epochs with *n* tracked satellites and resulting mean number of tracked satellites for a receiver offering 12 or more channels per antenna

*n*	Zenith antenna						Nadir antenna					
	GPS				Galileo		GPS				Galileo	
	L1 C/A	L2-CL	P(Y)	L5-Q	E1-C	E5a-Q	L1 C/A	L2-CL	P(Y)	L5-Q	E1-C	E5a-Q
0	0.0	0.3	0.9	0.0	0.0	0.0	0.0	0.0	0.0	0.0	0.0	0.0
1	2.0	0.9	14.3	1.2	1.8	0.0	0.1	0.0	0.7	0.0	0.0	0.0
2	10.3	3.8	27.5	9.4	14.2	0.5	0.2	0.0	5.6	0.4	0.0	0.0
3	24.2	19.5	34.9	14.4	40.0	11.6	2.8	1.5	17.6	1.5	0.6	0.0
4	36.1	46.1	18.8	22.3	36.4	40.5	10.3	5.8	30.4	8.0	3.4	0.0
5	23.1	27.9	3.5	28.2	7.6	36.4	29.0	15.0	25.8	24.2	14.0	0.0
6	3.4	1.6	0.0	17.4	0.0	10.0	28.8	28.9	15.5	22.4	29.6	3.4
7	0.9	0.0	0.0	5.8	0.0	1.1	18.5	26.5	2.9	14.1	27.9	15.5
8	0.0	0.0	0.0	1.1	0.0	0.0	8.2	15.3	1.4	13.7	18.7	34.9
9	0.0	0.0	0.0	0.0	0.0	0.0	1.7	5.8	0.1	9.5	5.0	28.3
10	0.0	0.0	0.0	0.0	0.0	0.0	0.3	1.1	0.0	4.8	0.8	13.6
11	0.0	0.0	0.0	0.0	0.0	0.0	0.0	0.0	0.0	1.5	0.0	3.9
12	0.0	0.0	0.0	0.0	0.0	0.0	0.0	0.0	0.0	0.0	0.0	0.4
Mean	3.8	4.0	2.7	4.5	3.3	4.5	5.8	6.5	4.4	6.5	6.6	8.5
Mean ($+{3}~\textrm{dB}$)	4.7	4.9	3.2	4.6	3.7	5.1	7.4	7.5	5.4	6.6	7.3	9.5
Mean ($-{3}~\textrm{dB}$)	2.9	3.3	2.3	3.9	3.0	3.9	5.0	5.4	3.2	6.1	5.8	7.5

The complementary $+{3}~\textrm{dB}$ and $-{3}~\textrm{dB}$ mean values refer to simulations with a corresponding increase or decrease in the simulated $C/N_0$ for all satellites

Based on preliminary specifications for the GENESIS GNSS subsystem, $C/N_0$ acquisition and tracking thresholds of 30 dBHz and 25 dBHz, respectively, were adopted in the visibility simulations for all openly accessible GPS and Galileo signals. For tracking of the GPS P(Y) signal, in contrast, we assumed reduced values of 15 dBHz and 10 dBHz that take into account the characteristics of common semi-codeless tracking implementations. Overall, a mostly monotonic variation of the signal quality with the off-boresight angle *z* of the receiving antenna is obtained due to the correlation of distance and receive/transmit antenna off-boresight angles for the specific antenna orientations (Fig. 5). Due to differences in transmit power and gain patterns among the various satellite types, some scatter in $C/N_0$ values at a given elevation can be recognized across the two constellations. Smaller-than-average values apply, for example, for the GPS Block IIR-A satellites and the Galileo IOV satellites.

Particularly, high signal strengths with peak $C/N_0$ values of about 58 dBHz are obtained near zenith due to the lower-than average distance. Given the limited, 24 dBHz, cross-correlation separation of the GPS C/A code, increased acquisition and tracking thresholds or aiding with predicted Doppler shifts may be required to avoid false locks due to cross-correlation for this signal. For the nadir antenna, tracking is limited to off-boresight angles larger than roughly $30^\circ$ due to Earth obstruction as already discussed in Sect. 2.

Signals in the lower L-band (i.e., L2, L5/E5a) mostly offer an increased $C/N_0$ at low elevation due to the wider transmit antenna beamwidth as compared to L1/E1. However, this benefit cannot be materialized when requiring dual-frequency measurements for compensation of ionospheric range delays. Based on top-side electron densities predicted by the NeQuick-G ionosphere model (EC 2016), observations with the zenith antenna may still be affected by slant total electron contents (STECs) at the level of $1 ~\hbox {TECU}$ or $10^{16}~{{\hbox {e}}^{-}/{\hbox {m}^{2}}}$ despite the large altitude of GENESIS. This results in range delays and phase advances on the order of one wavelength, which are clearly non-negligible in precise positioning and orbit determination. Even worse conditions with STEC values up to the 100 TECU level apply for the nadir antenna, thus clearly necessitating dual-frequency measurements for the GENESIS mission.

Focusing on the upper L-band, Galileo FOC satellites can be tracked in our simulations up to off-boresight angles of about $68^\circ$ with the zenith antenna and about $65^\circ$ with the nadir antenna, before passing below the adopted $C/N_0$ tracking threshold. Slightly higher values can be reached when tracking the two FOC satellites in eccentric orbits, while the reduced power levels of the IOV satellites result in a cut-off near $55^\circ$. In the case of GPS, semi-codeless P(Y) tracking (on L1 and L2) fails above $55^\circ$ to $60^\circ$ off-boresight angle under the current link-budget assumptions, which limits the use of old Block IIR-A/B satellites without L2C signal for GENESIS orbit determination. L1 C/A tracking, on the other hand, remains available up to about $85^\circ$ with the zenith antenna and about $70^\circ$ with the nadir antenna. This can mainly be attributed to the individual sidelobe peaks in the GPS antenna gain. For Galileo, details of the sidelobe gain patterns are not presently available, which may result in slightly conservative assumptions for the availability of low-elevation E1 tracking in the present simulation.

The overall statistics for the frequency of tracked satellites obtained with the two antennas for the individual GNSS constellations and signals are collated in Table 3. Compared to common low Earth orbits with altitude of less than 1500 km, a dramatically lower number of GNSS satellites can be tracked with a zenith pointing antenna from the GENESIS orbit. On average, only 3 to 4 GPS or Galileo satellites exhibit signal strengths above the assumed $C/N_0$ threshold. However, an almost two times higher number is accessible with the nadir-looking antenna in good accord with the general findings of Stanton et al. (2006) in a purely geometric GNSS visibility study for spacecraft in the SSV. Overall, an average total of ten tracked satellites per GNSS can be achieved with a dual-antenna configuration, which is well comparable with single-antenna tracking in a low Earth orbit.

Considering the increased hardware complexity of a dual-antenna GNSS system and prevailing limitations in the total number of tracking channels available in current space receivers, alternative boresight orientations may be considered. By way of example, a single antenna oriented “sideways” along the spacecraft $-x$-axis would, on average over all epochs, enable tracking of L1 C/A signals from 7.5 GPS satellites as well as E1-C signals from 5.7 Galileo satellites in the current simulation. While this provides only a small gain in terms of the average number of tracked satellites compared to the nadir-only antenna configuration, it offers a better geometric strength of the resulting observations as will be discussed in more detail in Sect. 5.

In addition to the mean number of tracked satellites for the nominal simulation conditions, Table 3 also provides the corresponding values for simulations with a ${3}~\textrm{dB}$ in- or decrease in the simulated $C/N_0$. This value reflects a reasonable worst case margin for the prevailing uncertainties in the transmit antenna gain and power as well as the receiver system noise and losses for the GENESIS mission. With limited exceptions for individual signals, it results in a 0.5 to 1.0 change in the mean number of tracked satellites.

## Performance characterization

Orbit determination of the GENESIS spacecraft is expected to be performed in a dynamic or reduced-dynamic (Wu et al. 1991) processing scheme along with the global adjustment of GNSS orbits, station coordinates, and Earth rotation parameters and combining all four space-geodesy techniques (Pollet et al. 2023). The dynamical orbit determination benefits from the physical constraints of orbital motion (Colombo 1989) and allows to obtain smooth and continuous orbit information even with sparse observation data. Furthermore, the dynamic orbit modeling is sensitive to the gravity field and can thus provide information on the location of the Earth’s center of mass.

On the other hand, the capability of aligning the reference frames from different space geodesy techniques will largely depend on the geometric information content of the respective measurements. In the case of GNSS with multiple concurrent code and phase pseudorange observations, even a purely kinematic orbit determination can be accomplished (Švehla and Rothacher 2003), which links the spaceborne GNSS observations to the terrestrial reference frame as realized by a global network of GNSS reference stations. By comparing the quality of kinematic GNSS position solutions of GENESIS with those of low Earth orbit (LEO) missions, we may thus obtain first insight into the contribution that GENESIS may provide for the improvement of the GNSS-based reference frame on top of existing LEO satellites.

**Table 4:** Statistics (mean value ± standard deviation) of horizontal and vertical dilution of precision for different configurations of GENESIS dual-frequency GNSS tracking

Case	GPS (L1 CA / L2-CL)				Galileo (E1-C/E5a-Q)				GPS+Galileo			
	HDOP		VDOP		HDOP		VDOP		HDOP		VDOP	
GENESIS (zenith)	–	(18%)	–	(6%)	–	(36%)	–	(11%)	–	(77%)	–	(42%)
GENESIS (nadir)	–	(75%)	–	(60%)	$1.3\pm 0.8$		$3.7\pm 1.5$		$0.9\pm 0.2$		$2.6\pm 1.0$	
GENESIS (combined)	$2.0\pm 1.3$		–	(80%)	$1.2\pm 0.5$		$3.5\pm 1.4$		$0.8\pm 0.1$		$2.4\pm 0.7$	
GENESIS (comb., +3 dB)	$1.4\pm 0.8$		$3.5\pm 1.5$		$1.0\pm 0.3$		$2.8\pm 1.2$		$0.7\pm 0.1$		$1.8\pm 0.5$	
GENESIS (comb., -3 dB)	–	(84%)	–	(53%)	$1.5\pm 1.0$		–	(92%)	$0.9\pm 0.3$		$3.2\pm 1.1$	
GENESIS ($-x$)	$3.1\pm 1.3$		$1.3\pm 0.8$		–	(83%)	–	(85%)	$1.7\pm 0.5$		$0.7\pm 0.3$	
Sentinel-6A	$1.8\pm 0.6$		$3.6\pm 1.1$		$1.8\pm 0.9$		$3.3\pm 0.8$		$1.1\pm 0.2$		$2.1\pm 0.4$	
LEO (zenith, 2x12 chan.)	$1.1\pm 0.3$		$2.2\pm 0.5$		$1.1\pm 0.3$		$1.9\pm 0.6$		$0.7\pm 0.1$		$1.3\pm 0.2$	

Only epochs with DOP values of less than 10.0 are considered. For configurations enabling a position solution with DOP<10 at less than 95% of all epochs, the corresponding availability is given instead. In addition to the simulated GENESIS configurations, DOP statistics for Sentinel-6A and a simulated LEO receiver supporting 12 dual-frequency channels per constellation are given for comparison. Except for the Sentinel-6A results, which are based on actual flight data with a $10^\circ$ elevation mask, a $0^\circ$ cut-off-angle was assumed in the remaining test cases

### Dilution of precision

Considering uniform weighting and identical measurement errors for all observations at a given epoch, the covariance of the positioning errors may conveniently be expressed as the product of the dilution of precision (DOP) and the standard deviation of the measurement errors (Fang 1981; Misra and Enge 2011). While originally intended for pseudorange-based positioning, the DOP concept may likewise be applied for carrier-phase based kinematic positioning, if we neglect the small contribution of uncertainties in the globally adjusted ambiguity parameters to the epoch-wise position errors.

In the case of a dual-constellation, dual-antenna receiver as considered for the GENESIS mission a total of four distinct receiver clock offset parameters $cdt_\text {GA}$, $cdt_\text {EA}$, $cdt_\text {GB}$, and $cdt_\text {EB}$ will need to be adjusted at each epoch next to the position $\varvec{r}$ to allow for the general case of time-varying inter-system biases between tracking of GPS (G), and Galileo (E), see Montenbruck et al. (2021), as well as time-varying biases between measurements from antennas A and B. Denoting by $\varvec{e}$ the line-of-sight unit vector from the receiver to a tracked GNSS satellite, the cofactor matrix

7 $$\begin{aligned} \varvec{C} = (\varvec{G}^T\varvec{G})^{-1}, \end{aligned}$$

i.e., the covariance for range errors of unit standard deviation, can then be expressed in terms of the design matrix

8 $$\begin{aligned} \varvec{G} = \left( \begin{array}{ccccc} -\varvec{e}^T_{1,\text {GA}} &{} 1 &{} 0 &{} 0 &{} 0 \\ \vdots &{} \vdots &{} \vdots &{} \vdots &{} \vdots \\ -\varvec{e}^T_{n_\text {GA},\text {GA}} &{} 1 &{} 0 &{} 0 &{} 0 \\ -\varvec{e}^T_{1,\text {EA}} &{} 0 &{} 1 &{} 0 &{} 0 \\ \vdots &{} \vdots &{} \vdots &{} \vdots &{} \vdots \\ -\varvec{e}^T_{n_\text {GB},\text {GB}} &{} 0 &{} 0 &{} 1 &{} 0 \\ -\varvec{e}^T_{1,\text {EB}} &{} 0 &{} 0 &{} 0 &{} 1 \\ \vdots &{}\vdots &{} \vdots &{} \vdots &{} \vdots \\ -\varvec{e}^T_{n_\text {EB},\text {EB}} &{} 0 &{} 0 &{} 0 &{} 1 \end{array} \right) , \end{aligned}$$

which describes the pseudorange partials with respect to the position and clock offset parameters for $n_{ij}$ tracked satellites of constellation $i=G,E$ and antenna $j=A,B$. For single-antenna or single-constellation tracking, a corresponding expression with a reduced number of estimation parameters and a reduced number of columns in Eq. (8) applies. Irrespective of the number of antennas and GNSSs, the horizontal and vertical dilution of precision are obtained as

9 $$\begin{aligned} \begin{array}{lcl} \text {HDOP} = \sqrt{C_{2,2}+C_{3,3}} \\ \text {VDOP} = \sqrt{C_{1,1}} \\ \end{array} \end{aligned}$$

from the leading diagonal elements of the unit-error covariance matrix, when expressing the line-of-sight vectors in a reference frame aligned with the radial, along-track, and cross-track direction.

Based on the previously described simulations, DOP statistics for the GENESIS mission have been determined considering various GNSSs and antenna configurations (Table 4). For GPS, availability of L1 C/A and L2-CL observations is assumed to enable dual-frequency positioning, while E1-C and E5a-Q availability is considered for the Galileo contribution. Given the fact that L2C is not yet supported by all satellites of the GPS constellation, this results in slightly conservative simulation results as compared to the targeted operations phase (2027++) of GENESIS, but is not considered to affect the overall conclusions that can be drawn from this analysis. Concerning GPS L5, an even lower availability applies and a minimum operational capability of 24 satellites transmitting L5 signals is presently only expected in 2029 (DOD/DOT/DOHS 2021). While attractive in view of the commonality with the Galileo E5a, use of this signal is not specifically considered in the DOP analysis. If indeed selected for the GENESIS receiver, a limitation in the number of GPS satellites supporting dual-frequency tracking similar to the current L2C availability would have to be expected. P(Y) tracking, on the other hand, is not further considered here due to the limitations of semi-codeless tracking at low signal strengths and an associated reduction in the number of tracked satellites (see Table 3).

In accord with the earlier link-budget and visibility analysis, tracking with only the zenith antenna would not enable an adequate positioning performance and deliver unacceptable DOP values for more than 50% of the time, even if considering combined GPS+Galileo tracking. Combined use of the zenith and nadir antenna offers good DOP values for the dual-constellation case and even reasonable performance for single-GNSS processing. However, it may be noted that very similar DOP values are achieved with only the nadir looking antenna. This may appear surprising in view of the fairly restricted off-boresight angle range covered by that antenna, but is partly explained by the fact that our DOP analysis assumes the estimation of independent, epoch-wise clock offset parameters for each antenna. As such, the joint availability of observations from both below and beneath the GENESIS spacecraft does not facilitate a better separation of the vertical position component and the clock offset parameter in the positioning compared to a single-antenna configuration.

Obviously, the DOP analysis does not account for the potentially different measurement and model errors of observations collected with the two antennas, but suggests that the proposed concept of a dual-antenna system requires further consideration. Based on Table 3, a total of about 8 dual-frequency channels would be required per constellation for tracking with only the nadir antenna, while an additional 6 dual-frequency channels per GNSS would be required to also cover the set of satellites accessible with the zenith antenna. While the overall number of tracking channels required for a dual-antenna receiver in the GENESIS mission is well supported by geodetic receivers for terrestrial applications, it would, for example, exceed the capacity of currently available radiation-hardened space receivers based on a single AGGA-4 correlator chip (Roselló et al. 2012) with 36 tracking channels. A careful trade-off between instrumental complexity and scientific benefit is therefore recommended for the mission.

In this context, it is also instructive to consider alternative antenna placements with different viewing directions. In analogy with the earlier visibility analysis, Table 4 includes results for a single-antenna configuration with a $-x$ boresight direction in the spacecraft body system. While the resulting sky view depends on the instantaneous yaw angle and varies notably throughout an orbit, the side-looking antenna would be able to simultaneously capture signals from satellites above and below the GENESIS spacecraft at the expense of a restricted coverage of the horizontal plane. In the first instance, this results in a swap of the relative HDOP and VDOP magnitudes, but also yields an obvious reduction in the overall position dilution of precision. The $-x$ antenna placement would therefore render itself as an interesting alternative, if a single-antenna configuration was adopted for the mission. In particular, the increased geometric strength of GNSS tracking in the radial direction offered by this choice may facilitate the validation of radial accelerations in orbit dynamics models and contribute to an improved determination of the GNSS-based TRF scale.

**Table 5:** Tracking-noise standard deviation in GENESIS and LEO simulations with nominal link budgets for pseudorange (PR) and carrier phase (CP) measurements

Case	GPS				Galileo			
	L1 C/A		L2-CL		E1-C		E5a-Q	
	PR (m)	CP (mm)	PR (m)	CP (mm)	PR (m)	CP (mm)	PR (m)	CP (mm)
GENESIS (zenith)	1.35	2.8	1.18	3.2	0.37	1.9	0.32	2.9
GENESIS (nadir)	1.21	2.5	1.22	3.3	0.45	2.3	0.35	3.1
GENESIS (-x)	1.39	2.9	1.19	3.3	0.49	2.6	0.38	3.4
LEO (zenith)	0.67	1.4	0.63	1.7	0.26	1.4	0.16	1.4

Noise statistics are computed from the full set of tracked satellites and represent a mix of observations collected at different elevations and $C/N_0$ values

Given prevailing uncertainties in the link-budget assumptions, we also investigated the impact of a 3 dB $C/N_0$ increase or decrease on the resulting DOP values. In this context, we emphasize that the DOP values are only affected by the varying number of satellites that can be acquired and tracked about the adopted thresholds, but are fully independent of the associated change in tracking noise implied by the $C/N_0$ in- or decrement. As shown in Table 4 for the example of the combined antenna configuration, the HDOP is only affected at the 10% level, which reflects a good distribution of tracked satellites over all azimuth angles independent of the signal power level. VDOP, on the other hand, is more sensitive to the achieved range of elevation angles and responds with a change of 20–30% to the ${\pm 3 ~\hbox {dB}}$ $C/N_0$ variation.

Finally, we compare the GENESIS DOP simulations with actual DOP results for the current Sentinel-6A mission, which uses a single, zenith-pointing antenna and operates in a low Earth orbit well within the Terrestrial Service Volume. Along with this, we also consider a simulated receiver in the same orbit, but offering more tracking channels and able to track all satellites in the antenna field of view down to the antenna horizon. While the latter configuration clearly exceeds the performance of all other test cases, the GENESIS mission can be expected to offer DOP values close to the actual Sentinel-6A mission under the nominal simulation assumptions. This confirms the overall feasibility of GNSS use in the adopted medium Earth orbit, but likewise suggests that only limited benefits of GENESIS for the GNSS-specific TRF realization can be expected compared to that of existing LEO missions.

### Measurement and model errors

The DOP analysis presented above describes the impact of the geometric distribution of tracked satellites and specifies the resulting positioning uncertainty for a unit ranging error. Statistical values of the overall position errors are then obtained by multiplication of the DOP with the expected standard deviation of the aggregate measurement and modeling errors. While it is difficult to fully specify the carrier phase error budget for the GENESIS mission at this stage due to major uncertainties in various system elements, we aim to provide at least a general discussion of the key contributions and expected range of magnitudes.

Except for the zenith antenna tracking, which benefits from the reduced GNSS satellite distance, GENESIS operates at systematically reduced $C/N_0$ levels compared to a receiver in LEO. As such, an increased level of thermal noise in the code and carrier phase observations is to be expected. For a given tracking loop bandwidth, measurement noise changes essentially by factor of two for a 6 dB change in $C/N_0$ (Betz 2015; Misra and Enge 2011) and would thus be 2–4 times higher than, for example, in Sentinel-6A when considering similar receiver characteristics.

Based on the theory of code and phase tracking errors due to thermal noise as a function of $C/N_0$ (Ward 2017) and receiver parameters representing the characteristics of the Sentinel-6A GPS/Galileo receiver, the overall standard deviation of pseudorange and carrier phase measurements has been computed based on the discussed simulations. Results for selected signals and test cases are summarized in Table 5. The various GENESIS antenna configurations result in fairly similar noise levels which are roughly twice as large as those of a corresponding LEO receiver as a result of the reduced average signal strength. Considering the ionosphere-free combinations of dual-frequency observations, a carrier phase noise of 8–10 mm is obtained. This translates into kinematic position errors with a standard deviation of about 15–30 mm for the DOP values of joint GPS/Galileo processing in Table 4, which was independently verified by processing of simulated code and phase observations with $C/N_0$-dependent noise in kinematic orbit determination with the Bernese GNSS Software (Dach et al. 2015) and the GNSS High precision Orbit Determination Software Tools (GHOST; Wermuth et al. 2010). In view of its white noise characteristics, the increased thermal noise level of the GENESIS mission is not concerning, though, and is not expected to degrade the orbit determination performance relative to established LEO missions in a significant manner.

On the other hand, various sources of systematic errors may be envisaged that impact the overall error budget. Irrespective of the high altitude of GENESIS, observations collected with the nadir and side-looking antenna may still pass the Earth’s atmosphere, which needs to be taken into account in the measurement modeling. While Earth-grazing signals passing through the neutral atmosphere can safely be discarded without relevant impact on the number of available observations, unmodeled higher-order ionospheric (HOI) effects may affect observations with tangent point altitudes of up to about 1000 km, or, equivalently, off-nadir-angle between $31^\circ$ and about $37^\circ$. Studies of LEO-based GNSS observations with zenith pointing antennas suggest representative HOI contributions below 1 cm even for very low orbital altitudes (Guo et al. 2023), and a similar magnitude is reported in Hernández-Pajares et al. (2014) for terrestrial receivers. Given the fact that Earth-grazing signals experience a two-times larger signal path through the ionosphere (GNSS to tangent point and tangent point to spaceborne receiver) than a terrestrial receiver, twice this magnitude can be expected in the case of the GENESIS mission for the given off-nadir angle range.

Another key source of systematic errors is given by unmodeled phase variations that remain after consideration of a priori values for the phase centers and patterns of both, receiver and transmit antennas. While pre-flight calibrations of GNSS receiver antennas for geodetic space missions are common practice by now, experience from numerous missions demonstrates notable deviations between the phase pattern of a stand-alone antenna calibration and the pattern observed after integration into the LEO satellite (Jäggi et al. 2009; Montenbruck et al. 2009; van den IJssel et al. 2015; Montenbruck et al. 2021). These deviations can be understood by short range multipath and distortions of the antenna field by neighboring spacecraft structures or occasional cross-talk between multiple GNSS antennas that are not considered in the ground calibration. Aside from a possible systematic phase center shift, patchy structures with amplitudes of 10–20 mm in the ionosphere-free dual-frequency combination are commonly observed in LEO missions, which depend on the LEO-to-GNSS line-of-sight direction and vary over representative scale lengths of $1^\circ$ – $30^\circ$. If uncorrected, such phase variations would induce root-mean-square (RMS) position errors at the few centimeter level similar to thermal receiver noise, but with a larger temporal and geographic correlation.

**Fig. 6:**
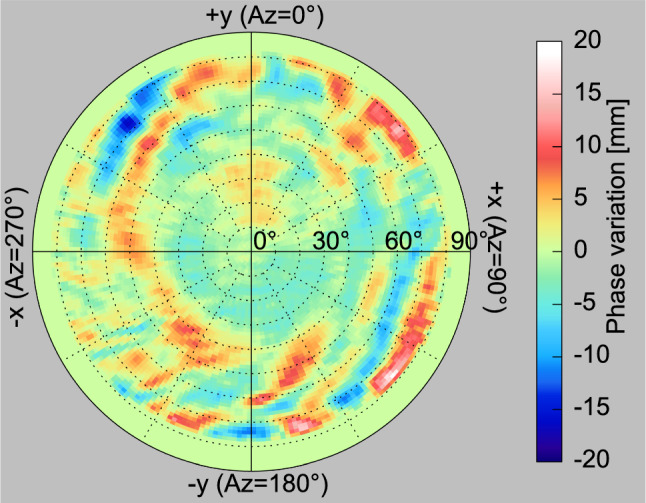
L1/L2 phase variations of the Sentinel-3A spacecraft derived from carrier phase residuals of ambiguity-fixed orbit determinations for March 24 to April 12, 2016

By way of example, phase pattern distortions observed in actual LEO missions are illustrated by the in-flight calibration of Sentinel-3A L1/L2 phase variations in Fig. 6, but similar patterns have been reported for other missions including, e.g., GRACE, GOCE, Swarm, Sentinel-1/2/6, and TerraSAR-X/TanDEM-X. Short-range phase variations clearly stand out in the residuals patterns observed in a dynamic or, to a lower extent, also a kinematic orbit determination. They can thus be estimated in an in-flight calibration and removed with remaining errors at the one or few millimeter level. However, estimation of phase patterns in the orbit determination is typically associated with a radial shift of the effective phase center location and therefore hampers an unambiguous, GNSS-based height estimation and a corresponding contribution to the TRF scale. To cope with this limitation, best efforts should be made to calibrate the phase patterns of all GNSS antennas as well as the DORIS and VLBI antennas for the GENESIS mission after complete integration of the entire spacecraft and, preferably, with deployed solar panels. While chamber calibrations for the entire spacecraft represent an obvious technical challenge, they appear as the only means to obtain a priori antenna phase patterns and phase center locations with 1-mm accuracy that will be required for meeting the GENESIS mission goals based on earlier E-GRASP simulations (Pollet et al. 2023).

A related challenge is provided by uncertain or incomplete information for the GNSS transmit antennas. Both GPS and Galileo satellites lack a comprehensive characterization of the transmit antenna phase patterns at high off-boresight angles, which introduces notable uncertainties in the carrier phase measurement model for GENESIS GNSS processing. In the case of Galileo FOC and IOV satellites, ground-calibrated transmit antenna phase patterns covering a range of up to $\theta =20^\circ$ and $\theta =14^\circ$, respectively, have been released by the system provider (ESA 2021), but no information for the Galileo transmit antennas is presently available beyond these limits. For GPS, phase patterns for GPS IIR-A/B, IIR-M, and III satellites up to $90^\circ$ off-boresight angle have been released by Lockheed Martin (Marquis 2015; Fisher 2022b) but refer to undocumented calibration reference points of the antenna panel rather than the spacecraft center of mass. For Block IIF satellites, phase information is included in the recently released manufacturer calibrations data (Igwe 2023), but lacks proper documentation and requires further processing and analysis before being accessible for geodetic processing.

For the Earth coverage zone, GNSS transmit phase patterns are generally flat with peak variations of less than 10 mm relative to the mean phase center. Comparisons with estimated phase patterns from terrestrial receiver networks and selected LEO missions (Dilssner et al. 2016; Conrad et al. 2023; Dilssner et al. 2023) show consistency at the one- to few-millimeter level with the Lockheed Martin antenna calibrations after adjustment of the individual phase center contributions. For $14^\circ \le \theta \le 17^\circ$, the lack of adequate LEO antenna patterns has resulted in IGS-specific, relative extensions of the GPS transmit antenna phase patterns (Jäggi et al. 2010) that are not, however, representative of the absolute patterns and exhibit deviations of up to 15 mm from the factory calibrations in this region.

**Fig. 7:**
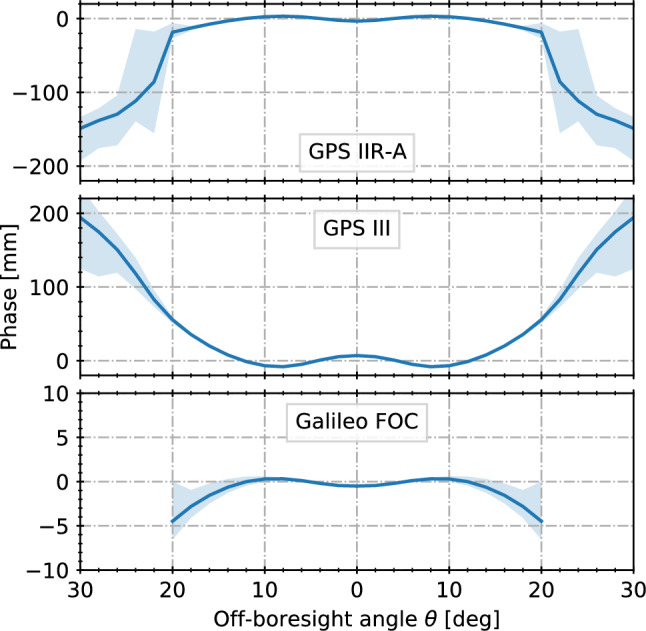
Azimuth-averaged L1/E1 phase variations of representative GPS IIR-A, GPS III, and Galileo FOC satellites (solid lines) as obtained in manufacturer calibrations. Shaded areas describe the range of phase variations at a given off-boresight angle for different azimuth angles. Note the different scales. All phase variations refer to phase centers minimizing the variation over the Earth coverage zone

For larger off-boresight angles, manufacturer calibrations indicate substantial differences in flatness among different transmit antenna types (Fig. 7). While Galileo FOC antennas exhibit phase variations of less than 10 mm within the currently published range of $20^\circ$, the GPS III L1 phase variations cover a range of roughly 70 mm below $\theta =20^\circ$, but almost 220 mm up to $30^\circ$ when referred to the currently adopted IGS phase center locations. Azimuth variations are particularly large near and beyond the gain minimum marking the transition from the antenna mainlobe to the sidelobes. This is most obvious for the Block IIR-A antennas, which show a reasonable flatness of L1 phase patterns up to $20^\circ$ but exhibit variations up to 200 mm beyond this range.

A need to independently determine those patterns as part of the GENESIS mission itself is therefore expected. Other than for the Earth coverage region, azimuth averaged patterns are no longer considered appropriate at high off-boresight angles, and the quality of such phase pattern estimates will crucially depend on the feasibility of using block-specific rather than satellite specific patterns. Only the zenith antenna or, alternatively, a side-looking antenna onboard the GENESIS spacecraft would offer joint access to observations within and above the Earth coverage zone and could thus enable phase pattern extensions to large off-boresight angles consistent with current IGS antenna models. For a stand-alone nadir antenna configuration, in contrast, GNSS phase patterns estimates would only cover off-boresight angles beyond $14^\circ$ and $12.5^\circ$ for GPS and Galileo, respectively, and thus, lack a proper connection with the established antenna patterns. A discussion of this limitation is beyond the scope of the present study, but would need to be assessed in a dedicated study of combined GNSS and GENESIS orbit, clock, and phase pattern estimation from terrestrial and spaceborne observations.

## Summary and conclusions

The GENESIS mission aims to enable a major improvement in the accuracy and consistency of terrestrial reference system realizations of individual space geodetic techniques. To achieve this goal, GNSS, DORIS, SLR, and VLBI instruments will, for the first time, be co-located on a single spacecraft. A compromise between the conflicting conditions of VLBI and GNSS tracking at different orbit height motivates the choice of a medium Earth orbit of 6000 km altitude. Compared to common remote sensing and geodesy satellites in low Earth orbits of 400–1400 km altitude, the much higher orbit of GENESIS implies widely different conditions for GNSS signal reception, which can partly be alleviated by the use of a dual-antenna system enabling observation of GNSS satellites in both, the zenith-looking and nadir-looking hemisphere.

To assess the conditions for GNSS tracking for GENESIS in a region well outside the Terrestrial Service Volume, detailed link-budget computations are performed to predict the expected $C/N_0$ and measurement noise levels. For this purpose, transmit antenna gain patterns for all current blocks of GPS and Galileo satellites have been compiled based on published manufacturer data or inferred from existing data of related antennas. These gain patterns are complemented by signal-specific transmit power values derived from EIRP measurements of a high-gain dish antenna along with modulation-specific power sharing ratios for the individual signals. Along with representative values for the receiver noise temperature and losses derived from a combined GPS/Galileo receiver currently in use onboard the Sentinel-6A satellite, detailed predictions of tracked satellites can thus be obtained for each of the two GENESIS antennas.

While the zenith antenna provides a good sky visibility, GNSS tracking is notably restricted by the limited beamwidth of the transmit antenna mainlobes. In particular, this limitation affects the L1 and E1 band, and results in a mean value of about four tracked satellites with dual-frequency observations per GNSS over all epochs. In accord with expectations for GNSS use in the space service volume, a notably better coverage with a mean of 6–7 tracked satellites per GNSS is obtained for the nadir-looking antenna. However, the respective signals are transmitted by the outer part of the GNSS transmit antenna mainlobe as well as partly the sidelobes and achieve a notably smaller peak signal strength than obtained with the zenith antenna.

Irrespective of this limitation, the nadir-antenna tracking dominates the resulting dilution of precision budget, which is only marginally improved when considering joint tracking with both antennas. Given comparable average $C/N_0$ values from both antennas and the improved geometric information content, use of only the nadir antenna may be considered as an option for the GENESIS mission, to minimize the complexity of the GNSS instrumentation. As an alternative, use of a side-looking antenna may be considered, which enables an even better position DOP and minimizes the vertical DOP component. Overall, DOP values for GENESIS are found to be to roughly comparable with existing LEO missions, even though the latter could readily be improved by up to a factor of two, when allowing for a larger number of tracking channels and tracking down to the antenna horizon.

Given comparable DOP values, but increased measurement noise and phase pattern uncertainties, the kinematic orbit determination performance of GENESIS using stand-alone GNSS is currently expected to fall behind that achievable with current LEO satellites. More detailed simulation of combined orbit determination and global parameter adjustment for GENESIS and the GNSS constellations will therefore need to be performed to further evaluate the benefit that GENESIS will be able to offer for the alignment of technique-specific reference frame realizations as compared to existing missions (such as Sentinel-3A, -3B and -6A) with GNSS, DORIS, and SLR co-location (Schreiner et al. 2023). While GENESIS provides the first opportunity to complement these techniques with VLBI and thus offers a comprehensive set of space ties, dedicated efforts will be required to improve the pre-flight calibrations of all instruments over current LEO missions, when aiming at a quantitative improvement of reference frame accuracy and consistency.

Concerning the GNSS contribution of GENESIS, the assessment of the expected tracking coverage and the kinematic orbit determination performance presently suffers from an incomplete characterization of the GNSS transmit antennas. Despite a partial release of metadata for the GPS and Galileo satellites, major gaps remain in the understanding of gain and phase patterns beyond the Earth coverage region for various satellite and antenna types. However, sidelobe characteristics are known to have vital impact for the availability of GNSS tracking at high altitude and proper understanding is likewise desired to ensure optimum performance of GNSS tracking in GENESIS. As such, a comprehensive public release of pre-flight calibrations by GNSS providers or satellite manufacturers is strongly desired and encouraged.

## Data Availability

GNSS transmit antenna patterns used for the simulation are available in the public literature as given in the list of references. Public access to Sentinel GNSS observations is provided at the Physical Oceanography Distributed Active Data Center (PODAAC; https://podaac.jpl.nasa.gov/) and the precise orbit determination hub (https://scihub.copernicus.eu/gnss) of the Copernicus program.
